# Performance analysis using the classification composition and match records in wheelchair basketball matches

**DOI:** 10.3389/fspor.2025.1542023

**Published:** 2025-04-28

**Authors:** Seunghun Lee, Min-Chang Kim

**Affiliations:** Institute of Disability Sport Science, Korea National Sport University, Seoul, Republic of Korea

**Keywords:** wheelchair basketball, performance, classification, para sports, trend analysis, team sports

## Abstract

**Introduction:**

This study provides essential information for wheelchair basketball coaches and players to enhance tactical applications and training for improved performance. By examining the latest trends in sports classification and performance factors influencing game outcomes, this study presents a comparative analysis across different levels of international wheelchair basketball play.

**Methods:**

To achieve this objective, major game factors were examined by analyzing descriptive statistics from each year regarding recent trends in sports class composition and the playing time of each class, followed by group difference tests. A total of 209 official game records from 24 teams participating in major international wheelchair basketball tournaments were analyzed. Group differences were tested in terms of sports class composition, playing time, and performance metrics.

**Results:**

First, scoring factors directly affecting game results were compared between groups. The difference test showed that the success rates of 2-point (50.73%) and 3-point (31.41%) shots differed significantly, while the free throw success rate did not. Significant differences were also found in the number of assists (22.94), defensive rebounds (27.38), and steals (5.95). Second, the medal group was compared with the non-medal group. The average sports class composition per quarter was significantly higher in the medal group (1QSC: 14.00, 2QSC: 13.96, 3QSC: 13.98, 4QSC: 13.96) than in the other group (1QSC: 13.89, 2QSC: 13.89, 3QSC: 13.85, 4QSC: 13.88). In terms of playing time differences by class, medal group players showed longer participation: 2.5-point (22:21), 4.0-point (14:46), 3.0-point (19:05), and 1.5-point (16:15). Third, from 2012 to 2022, trends in sports class composition and quarterly playing time have evolved. In 2022, the average playing time of 1.5-point and 4.0-point athletes decreased by about 4 min compared to 2012, while the playing time of 4.5-point athletes increased by approximately 5 min and that of 3.0-point athletes increased by about 2 min.

## 1 Introduction

Wheelchair basketball (WB) is one of the most popular official events in the Paralympic Games (PG). Since 1964, when it was selected as an official game event at the Tokyo PG, WB has spread worldwide for over 60 years. Furthermore, the PG are held quadrennially, while world championship games (WC) and regional international competitions continue to be held under the supervision of the International Wheelchair Basketball Federation (IWBF) to encourage para-athletes to participate and compete to achieve their potential.

**Figure 1:**
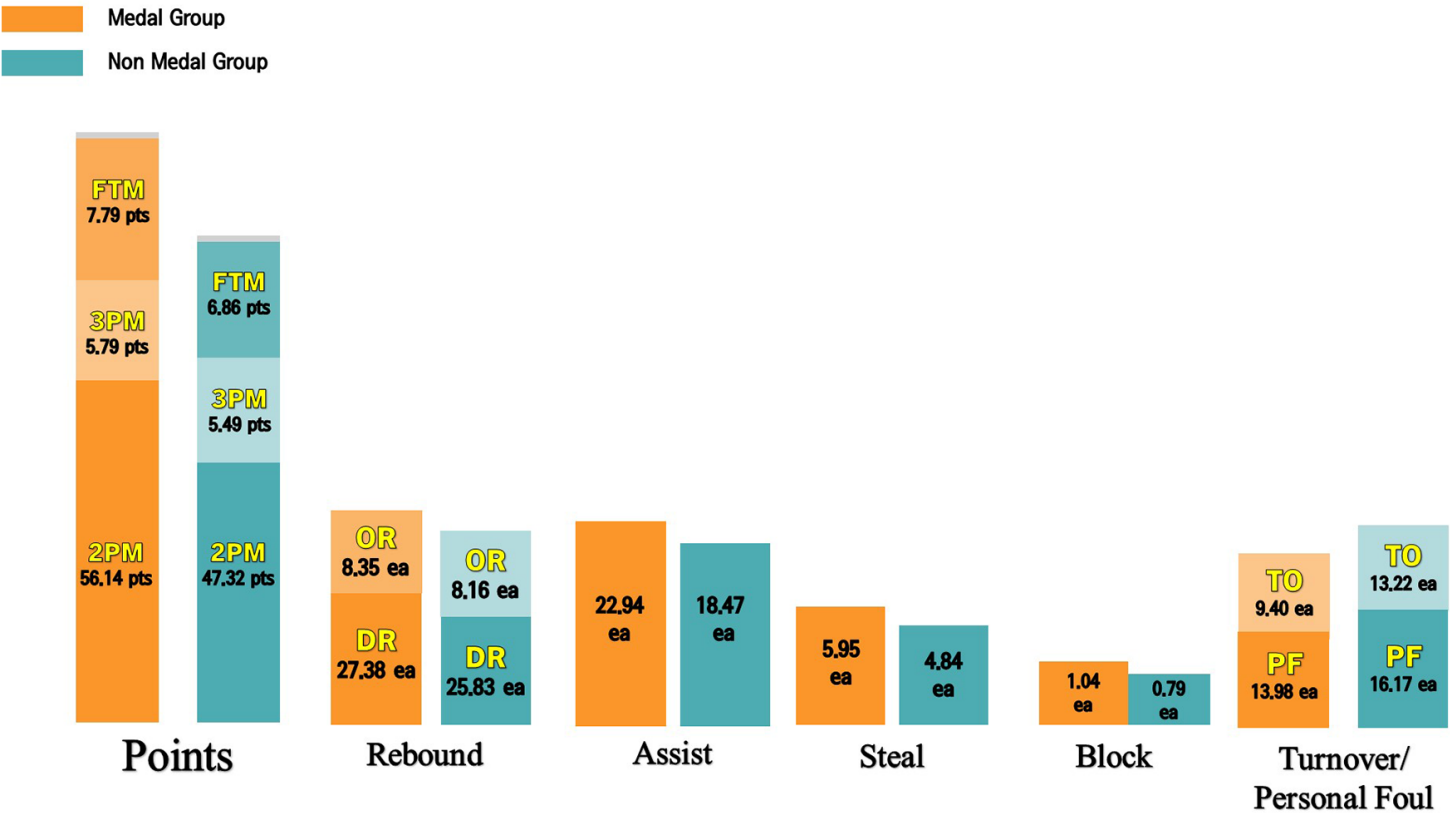
Game trend results from 2012 to 2022.

From 2012 to 2022, about 27 countries participated in the WC and PG of WB. As of 2024, members of the IWBF include 24 in Africa, 21 in America, 28 in Asia and Oceania, and 36 in Europe, with about 289 players from 109 countries. Wheelchair basketball leagues are actively conducted around the globe, including Europe, North America, Asia, and so forth (1).

As WB events have become common and developed, para-athletes' personal skills and team performances in WC and PG have improved significantly. As a result, global competitiveness is intense, with more diversified strategies required more than ever before in preparation for each competition (2).

Basketball games have a fast transition between offense and defense, requiring fast and delicate judgment because the game result may be changed within seconds. To secure winning and outstanding team performance, the optimized team of players is organized for each game (3). Particularly in contemporary basketball, roles are divided explicitly among 5 players for specific strategies and tactics, which definitely decide the victory and defeat of the game. An athlete's skill is essential in team sports, but the team's organization and tactics are also vital to winning (4–6).

Similarly, in WB, player selection can factor in winning. Still, due to the “Sports Classification System”, it has to be organized differently than in basketball. In particular, due to the decreasing number of international competition games and changes in the classification of para-athletes, the major national teams (the United States of America, Great Britain, Australia, etc.) are no longer relying on individual performance but on team cohesion and tactics to win matches. Furthermore, the overall trend of principal countries is to select the national team by identifying the appropriate combination of players and the sum of their classifications to maximize performance and teamwork. In other words, to formulate a strategy, match analysis of WB needs to assess the match factors that affect the match results, and the factor analysis related to each player's sports class points as a significant part of the team performance characteristics (5).

In general, basketball playing styles and roles change depending on international trends and training methods (7). For example, Štrumbelj et al. (8) points out that ever since 2001, when the shot clock changed from 30 s to 24 s, the numbers of team offenses, earning scores, and two-point shots have increased during the 10 seasons, whereas the number of 3-point shots has decreased. As the number of three-point attempts in international basketball increased in 2010, and as offensive and defensive transitions became faster, teams have demanded players to attack using space and defend in various patterns (9). Players are given multiple roles, and many different training methods are applied flexibly in line with international trends.

Similar to the changes in basketball mentioned above, WB has also seen changes in the rules and how the game gets played. For example, the sports classification in WB was changed to the evidence-based form in the 2016 Rio PG, and the new minimum impairment criteria were applied in the 2021 Tokyo PG. Factors affecting sports performance in para sports include participants' classification, health condition, and training method, among which classification factors vary significantly by a player's function. Therefore, understanding the sports classification factors and playing time among countries known for the central team's performance will be vital in deciding strategies.

Although such research is not relatively active in WB, the game operation is similar to that of ordinary basketball. Thus, strategies may be established based on similar analytic approaches for game performance improvement. Previous studies in WB focused on physical abilities and analyses of victory and defeat based on team records (10–13).

Additionally, sports class factors, a deciding characteristic for WB, are significant elements in analytic approaches for game performance improvement. However, previous studies on the sport class of WB focused on kinematics and differences in skills and physical functions related to each player's points in terms of sports medicine (14–18). As in previous studies, such analyses on individual players' physical functions and abilities have limitations in understanding major factors affecting WB game performance.

Given the limitations of previous research, as mentioned earlier, the association among sports performance, sports-class composition (classification), and match results need to be investigated. In previous studies, variables other than sports-class and playing time (offense, defense, turnovers, etc.) were used to analyze the difference in performance between the top and bottom groups (13, 16). However, since WB limits the number of points that can be played (within 14 points) to minimize the type of impairment that affects performance, it is important to compare the combination of points and playing time per quarter for each group. In addition, as of the Rio PG, the International Paralympic Committee introduced an evidence-based classification for each para sport (19), and some of the classes were adjusted in wheelchair basketball, which resulted in a tendency for the number of points to fluctuate. For this reason, this study aims to analyze the key factors influencing WB performance by examining national performance characteristics and the composition of sports classes, dividing players into high- and low-performing groups based on official records from central international men's WB competitions, by diagnosing the primary variables and identifying trends in sports class composition and performance factors.

Ultimately, this study provides essential information for WB coaches and players to enhance tactical application and training for improved performance. By examining the latest trends in sports classification and the performance factors that impact game outcomes, this study will present a comparative analysis across different levels of play in international WB.

## 2 Method

### 2.1 Data collection

This study analyzes the changes in “sport classification composition” and “playing time by classification” between high-performing and non-high-performing groups in WB. As the sport undergoes changes due to the recent application of evidence-based classification led by the IPC, the analysis explores key competitive variables to identify emerging trends.

To analyze the performance details of international wheelchair basketball games, this study selected the official records of 209 games among 24 teams that participated in major international wheelchair basketball games from August 30, 2012, to June 20, 2023 (*n* = 418). To achieve the objectives of this study, data were collected from the IWBF WC and PG held in London (2012) and Rio (2016) before the introduction of evidence-based classification, as well as from the IWBF WC and PG after the introduction of this classification. Additionally, as most research on the IWBF's rules and classification has been conducted since the 2010s, score sheets from the 2010 Games were likewise collected.

This study gathered data from publicly official records on the IWBF's website (https://iwbf.org/, accessed on 12 March 2024). Furthermore, the researcher obtained the sports class composition for each quarter through videos on the IWBF and FIBA's official websites and YouTube. However, as shown in Table 1, the researcher removed those that did not have uploaded videos or did not accurately display the official results.

**Table 1:** Number of 2012–2022 WC and PG.

Event	Number of games	Remark
2012 London PG	38	Not uploaded 6 games
2016 Rio PG	42	
2018 Hamburg Wheelchair Basketball WC	41	Not uploaded 7 games
2020 Tokyo PG	41	Not uploaded 1 games
2022 Dubai Wheelchair Basketball WC	47	Data missing 6 game
**Total**	209	

### 2.2 Data processing

First, descriptive statistics analysis was performed on collected match records with calculated average and standard deviation. To understand the differences between the groups that determine the level of competition in sports, this analysis distinguished between countries that won medals in each sport and those that did not. Medal-winning countries are fewer in number in each competition, but they represent superior performance and inspire other countries regarding strategy and tactics.

Second, the non-parametric statistics technique, “Mann–Whitney *U*-Test”, was performed to verify differences in the match results, sports class composition, and playing time (see Table 2) using the IBM SPSS 27.0 program. The Mann–Whitney *U*-test is a non-parametric test that compares the means of samples with the same population characteristics and determines the difference between two sample means (20). In this study, the Mann–Whitney *U*-test was applied to identify nonparametric differences in the number of medal-winning and non-medal-winning countries participating in the International Wheelchair Basketball Games and to identify nonparametric differences in the outcomes of competitions and the classification between groups. Since the difference in number between the medal group and the other group was significant and the basic assumption of parametric statistics (normality test) failed, the non-parametric statistics method was applied instead. The statistical significance level was set to.05.

**Table 2:** Research subjects and variables.

Subjects	Variables	Subjects	Variables
1QSC	1st quarter Sport Class Composition	2PA	2-Points Attempt
2QSC	2nd quarter Sport Class Composition	2P%	2-Points Shooting Percentage
3QSC	3rd quarter sport class Composition	3PM	3-Points Made
4QSC	4th quarter sport class Composition	3PA	3-Points Attempt
1.0 played minutes	1.0-point player played minutes	3P%	3-Points Shooting Percentage
1.5 played minutes	1.5-point player played minutes	FTM	Free Throw Made
2.0 played minutes	2.0-point player played minutes	FTA	Free Throw Attempt
2.5 played minutes	2.5-point player played minutes	FT%	Free Throw Percentage
3.0 played minutes	3.0-point player played minutes	OR	Offensive Rebounds
3.5 played minutes	3.5-point player played minutes	DR	Defensive Rebounds
4.0 played minutes	4.0-point player played minutes	TOT	Total Rebounds
4.5 played minutes	4.5-point player played minutes	AS	Assists
PTS	Points	TO	Turnovers
FGM	Field Goals Made	ST	Steals
FGA	Field Goals Attempt	BS	Block Shots
FG%	Field Goals Shooting Percentage	PF	Personal Fouls
2PM	2-Points Made		

Third, trends were analyzed in international wheelchair basketball games each year. Game trends were analyzed based on the descriptive statistics of annual match records. Specifically, trend analysis is used to identify the records of para-athletes and teams to check the performance contents of national athletes and to set target standards to promote performance in international sports competitions (19). Therefore, the trend information applied in this study can be used to compose the WB line-up and training by checking trends in the performance of principal countries in international wheelchair basketball competitions.

## 3 Results

### 3.1 Descriptive statistics

Descriptive statistics are presented in Table 3. As for results depending on the sports classes, sports classes competing in each quarter were given almost 14 points. The playing time of each sports class was 17:44 min for players of 1.0 points, 18:53 min for players of 1.5 points, 17:46 min for players of 2.0 points, 18:47 min for players of 2.5 points, 21:12 min for players of 3.0 points, 15:02 min for players of 3.5 points, 16:52 min for players of 4.0 points, and 17:55 min for players of 4.5 points. In general, players of 1.5 points, 2.5 points, and 3.0 points were given longer playing time than others. In a review of game results, the average score of each game was 61.94. The successful field shots and attempts were 26.50 and 59.94, respectively. The successful 2-point shots and attempts were 24.64 and 52.35, respectively. The successful 3-point shots and attempts were 1.86 and 7.61, respectively. The successful free throws and attempts were 7.09 and 12.35, respectively. The offense rebounds and defense rebounds per game were 8.22 and 26.22. The assists, steals, block shots, turnovers, and errors were 19.61, 5.13, 0.85, 12.25, and 15.60, respectively.

**Table 3:** Descriptive statistics on game (2012 to 2022).

Variables	N	Mean	SD	Variables	N	Mean	SD
1QSC	418	13.92	.224	2PA	418	52.35	7.353
2QSC	418	13.91	.228	2P%	418	46.71	9.127
3QSC	418	13.88	.280	3PM	418	1.86	1.637
4QSC	418	13.90	.280	3PA	418	7.61	4.344
1.0 played minutes	408	17:44	7:44	3P%	418	24.24	20.229
1.5 played minutes	250	18:53	8:53	FTM	418	7.09	4.284
2.0 played minutes	291	17:46	9:25	FTA	418	12.35	6.627
2.5 played minutes	290	18:47	9:43	FT%	418	55.74	19.522
3.0 played minutes	347	21:12	9:27	OR	418	8.22	3.719
3.5 played minutes	264	15:02	9:54	DR	418	26.22	5.440
4.0 played minutes	356	16:52	9:08	TOT	418	34.44	6.790
4.5 played minutes	339	17:55	8:44	AST	418	19.61	6.025
PTS	418	61.94	15.034	STL	418	5.13	3.162
FGM	418	26.50	6.585	BLK	418	0.85	1.140
FGA	418	59.94	6.544	TO	418	12.25	5.397
FG%	418	43.99	9.000	PF	418	15.60	4.602
2PM	418	24.64	6.511				

**Table 4:** Difference test depending on the quarterly sport class composition and playing time in groups.

Variables	Medal group		Non-Medal group		Mann–Whitney *U*	Sig
	Mean	SD	Mean	SD		
1QSC	14.00	.001	13.89	.255	13788.500	.001***
2QSC	13.96	.183	13.89	.241	14873.500	.003**
3QSC	13.98	.118	13.85	.310	13382.000	.001***
4QSC	13.96	.155	13.88	.309	14841.000	.004**
1.0 played minutes	16:37	7:53	18:08	7:39	14693.500	.152
1.5 played minutes	16:15	7:11	19:21	9:05	3106.000	.025*
2.0 played minutes	17:51	8:59	17:45	9:36	8193.000	.858
2.5 played minutes	22:21	9:21	16:49	9:24	6620.500	.001***
3.0 played minutes	19:05	11:19	22:04	8:27	9704.000	.002**
3.5 played minutes	14:55	8:08	15:05	10:29	6499.000	.522
4.0 played minutes	14:46	10:09	17:38	8:37	9652.500	.001***
4.5 played minutes	17:34	7:32	18:03	9:08	10748.500	.503

* *p* < .1.

** *p* < .05.

*** *p* < .01.

**Table 5:** Difference test on game results in groups (2012 to 2022).

Variables	Medal group		Non-Medal group		Mann–Whitney *U*	Sig
	Mean	SD	Mean	SD		
PTS	69.75	10.490	59.28	15.453	9700.500	.001***
FGM	30.01	4.715	25.29	6.725	9409.000	.001***
FGA	61.83	5.918	59.28	6.645	13034.500	.001***
FG%	48.58	6.293	42.42	9.278	9858.500	.001***
2PM	28.07	4.852	23.46	6.612	9464.500	.001***
2PA	55.35	6.102	51.30	7.462	11582.000	.001***
2P%	50.73	6.747	45.33	9.462	10927.000	.001***
3PM	1.93	1.500	1.83	1.687	15951.000	.401
3PA	6.49	3.697	8.01	4.482	13514.000	.002**
3P%	31.41	23.393	21.72	18.399	12586.500	.001***
FTM	7.79	4.436	6.86	4.210	14804.000	.060
FTA	12.95	6.536	12.17	6.660	15681.000	.284
FT%	57.64	17.788	55.17	20.061	15149.000	.118
OR	8.35	3.550	8.16	3.781	16398.000	.682
DR	27.38	5.286	25.83	5.456	13988.000	.008**
TOT	35.73	5.952	33.99	7.031	13906.000	.007**
AST	22.94	4.956	18.47	5.948	9525.000	.001***
STL	5.95	3.937	4.84	2.806	14242.000	.016*
BLK	1.04	1.380	.79	1.040	15390.000	.148
TO	9.40	3.701	13.22	5.552	9958.500	.001***
PF	13.98	4.937	16.17	4.343	12346.000	.001***

* *p* < .1.

** *p* < .05.

*** *p* < .01.

**Table 6:** The quarterly sport class composition trend and playing minutes from 2012 to 2022.

Year	2012		2016		2018		2020		2022	
	Mean	SD	Mean	SD	Mean	SD	Mean	SD	Mean	SD
1QSC	13.95	.247	13.92	.187	13.89	.261	13.96	.141	13.89	.253
2QSC	13.99	.081	13.92	.198	13.90	.269	13.90	.219	13.87	.286
3QSC	13.94	.182	13.91	.193	13.85	.280	13.90	.228	13.83	.412
4QSC	13.95	.193	13.90	.277	13.92	.215	13.90	.214	13.83	.405
1.0 played minutes	20:09	6:44	17:49	6:20	13:48	6:09	20:56	10:45	16:28	5:38
1.5 played minutes	17:56	8:20	17:54	6:56	15:59	10:07	21:15	9:11	21:25	8:36
2.0 played minutes	15:59	9:43	19:35	8:03	18:37	10:44	17:50	9:22	16:45	8:50
2.5 played minutes	18:02	7:07	20:11	7:29	16:35	10:06	21:31	10:34	17:25	10:48
3.0 played minutes	21:32	8:36	23:08	7:34	21:35	11:12	20:38	10:05	19:04	8:53
3.5 played minutes	11:31	6:39	16:44	9:11	16:36	14:01	16:08	8:37	14:10	7:35
4.0 played minutes	15:30	7:33	17:38	7:45	13:53	8:54	17:58	8:43	19:17	8:17
4.5 played minutes	22:16	7:37	18:03	8:24	13:24	7:21	19:22	8:34	17:09	9:13

**Table 7:** Game trend results from 2012 to 2022.

Year	2012		2016		2018		2020		2022	
	Mean	SD	Mean	SD	Mean	SD	Mean	SD	Mean	SD
PTS	62.79	12.596	61.62	14.702	62.62	11.847	61.65	12.080	61.18	21.011
FGM	26.63	5.396	26.32	6.632	26.94	5.201	26.56	5.444	26.11	9.055
FGA	59.68	5.951	58.94	6.489	60.09	5.782	62.13	6.061	58.99	7.656
FG%	44.67	8.211	44.38	8.782	44.83	7.782	42.68	7.491	43.49	11.641
2PM	25.41	5.315	24.23	6.751	25.11	5.214	24.45	5.416	24.15	8.736
2PA	54.63	5.501	50.62	7.832	52.63	5.929	53.95	6.350	50.40	9.187
2P%	46.49	8.488	47.38	8.918	47.66	8.386	45.17	7.954	46.79	11.169
3PM	1.22	1.184	2.10	1.767	1.83	1.464	2.11	1.757	1.96	1.759
3PA	5.05	3.233	8.32	4.443	7.45	3.411	8.18	3.676	8.69	5.420
3P%	24.17	26.033	24.45	17.171	24.35	18.666	24.46	16.862	23.81	21.769
FTM	8.30	4.336	6.88	3.698	6.91	4.284	6.44	4.006	7.01	4.827
FTA	14.61	6.999	12.01	6.083	11.77	6.270	11.34	6.149	12.20	7.191
FT%	56.40	16.559	58.31	18.699	55.27	21.607	53.97	20.536	54.85	19.791
OR	8.74	3.616	7.94	3.316	8.71	3.707	8.18	3.775	7.68	4.062
DR	26.71	5.659	26.44	5.389	26.12	4.623	28.67	5.159	23.55	5.140
TOT	35.45	6.898	34.38	6.577	34.83	5.370	36.85	6.466	31.23	7.191
AST	19.36	5.174	20.58	6.334	19.30	5.298	21.44	5.200	17.64	7.034
STL	3.53	2.306	5.67	3.469	5.66	3.183	5.39	2.792	5.28	3.390
BLK	1.11	1.014	1.30	1.612	0.67	1.101	0.72	0.836	0.51	0.786
TO	11.54	4.374	13.56	5.613	12.84	5.290	11.54	4.606	11.76	6.422
PF	17.63	4.738	16.08	4.314	14.60	3.820	15.23	4.392	14.73	5.038

### 3.2 Difference test

#### 3.2.1 Composition and playing time by sports class between groups

The second set of research findings is about the difference in match records between the groups from 2012 to 2022 in Tables 4, 5. Given the difference in test results between groups, particularly regarding the sport class, the medal group showed a higher level of points in the average quarterly rating (1Q: 14 points, 2Q: 13.96 points, 3Q: 13.98 points, 4Q: 13.96 points) than the other group (1Q: 13.89 points, 2Q: 13.89 points, 3Q: 13.85 points, 4Q: 13.88 points), and the difference was significant. In addition, the playing time depending on the points of the medal group was as follows: 2.5 points (22:21), 3.0 points (19:05), 2.0 points (17:51), 4.5 points (17:34), 1.0 points (16:37), 1.5 points (16:15), 3.5 points (14:55), and 4.0 points (14:46) in order. That of the other group was as follows: 3.0 points (22:04), 1.5 points (19:21), 1.0 points (18:08), 4.5 points (18:03), 2.0 points (17:45), 4.0 points (17:38), 2.5 points (16:49), and 3.5 points (15:05) in order. The difference in playing time between the medal group and the other group was in the order of 2.5 points (*p* = .001), 4.0 points (*p* = .001), 3.0 points (*p* = .002), and 1.5 points (*p* = .025). This shows that the difference in playing time was significant.

#### 3.2.2 Composition and playing time by sports class between groups

Given the results in Table 6, the success rates of 2-point and 3-point shots showed a considerable difference except for the free throw success rate. The most significant difference was observed in the 3-point shot success rate (*p* = .001): that of the other group was 1.83 out of 8.01 attempts, while that of the medal group was 1.93 out of 6.49 attempts. This means that the success rate of the medal group (31.41%) was 9.69% higher than that of the other group (21.72%). As for the success rate of 2-point shots (*p* = .001), the other group was 23.46 out of 51.30 attempts, while that of the medal group was 28.07 out of 55.35 attempts. This means that the success rate of the medal group (50.73%) was 5.4% higher than that of the other group (45.33%). In contrast, the free throw success rate of the other group was 6.86 out of 12.17 attempts, while that of the medal group was 7.79 out of 12.95 attempts. Thus, the success rate of the medal group (57.64%) was 2.47% higher than that of the other group (55.17%), which means that the difference is statistically insignificant. The average offense rebounds, defense rebounds, steals, and block shots in each WB game were 8.22, 26.22, 19.61, 5.13, and 0.85, respectively. As these records were comparatively analyzed regarding game performance between the medal group and the other group, the difference in assist, defense rebound, and steal was significant. First, the number of assists (*p* = .001) in the medal group (22.94) was 4.47 more than that in the other group (18.47), which is a significant difference. The number of defense rebounds (*p* = .008) in the medal group (27.38) was 2 points more than that in the other group (25.38), which is a significant difference. The number of steals (*p* = .016) in the medal group (5.95) was 1.11 points higher than that in the other group (4.84), which is a significant difference. The number of turnovers (*p* = .001) in the medal group (9.40) was 3.82 points less than that of the other group (13.22), which is a significant difference. The number of fouls (*p* = .001) in the medal group (13.98) was 2.19 points less than that of the other group (16.17), which is a significant difference.

### 3.3 Trend analysis

The third set of research findings is about the trend in descriptive statistics of match records from 2012 to 2022 in Table 7. As to changes depending on the sports class, the quarterly sports class composition tended to decrease. As to the playing time, depending on the range of points, that of 1.0 points, 2.5 points, 3.0 points, and 4.5 points decreased, while that of 1.5 points, 2.0 points, 3.5 points, and 4.0 points increased. As to the playing time, depending on the range of points, that of 1.0 points, 2.5 points, 3.0 points, and 4.5 points decreased, while that of 1.5 points, 2.0 points, 3.5 points, and 4.0 points increased. Notably, the playing time of WB players of a mild case (4.5 points) decreased by about 5:07 min. In contrast, the number of players with 3.5 and 4.0 points increased by as much as 3:09 min and 3:47 min, respectively.

As time passed, one competition after another, the general scores and numbers of field shots, 2-point shots, free throws, rebounds, assists, block shots, and personal fouls decreased. In contrast, 3-point shot success rates and numbers of attempts, steals, and turnovers increased.

Mainly, 3-point shot successes and attempts increased as much as 0.74 and 3.64, respectively, in 2022 compared to 2012. The 2-point shot success rate also increased. Steals increased by as much as 1.75 and turnovers by 0.22 in 2022 compared to 2012.

## 4 Discussion

This study provides valuable insights for WB coaches to develop tactics and for players to enhance their performance by examining the latest trends in sports classification and performance that influence international WB at different performance levels. To achieve the objective of this study, official records and video games of 209 PG and WC were collected, and 418 match records in total were analyzed, including the ratings for each country. Furthermore, a non-parametric test, the Mann–Whitney *U*-test, was applied to examine differences in performance between groups. Additionally, trend analysis was conducted to identify players' progression in playing time by sports classes and performance over time. Firstly, match records of IWBF games from 2012 to 2022 were analyzed, and the results are as follows: Scoring factors directly affecting the game results were compared between the groups (see Figure 1). The result shows that the success rate of 2-point and 3-point shots was significantly different except for the free throw success rate, and the numbers of assists, defense rebounds, and steals were significantly different. This result shows that the medal group used various offensive tactics that contributed to scoring and hindered the other team from having opportunities for secondary scoring through defense rebounds and quick transitions. Prior research in basketball game analysis has shown that modern basketball prefers a fast-paced, aggressive style. That play is strongly associated with higher success rates in three-pointers, steals, and rebounds (21–23). As suggested by the results of this study, the international WB events show similar trends to those of basketball. Furthermore, the game performance showed differences in terms of defense rebound and steal, which contribute to switching the other team's scoring attempts into our team's offense opportunities, as well as assists that are directly related to scoring, just as in basketball games (12, 23–25). The difference in turnovers and fouls was also significant between the outstanding and the other groups. In addition, the medal group recorded fewer turnovers and fouls than the other group. According to the research, turnovers increase the probability of giving the other team opportunities to win a score, and it is known that turnovers increase the likelihood for the other team to win a score and cut off the flow of our team's offense on turnovers (26–28). If a particular player has many fouls and needs to prepare for free throw opportunities thoroughly, the player can be an easy target for the other team to score. The result of this study also shows that the medal group recorded smaller numbers of turnovers and fouls than the other group. Thus, compared to the non-medal group, the performers are making and attempting more shots that directly affect scoring (2-points, 3-points, and free throws), and they have a higher frequency of defensive rebounds and steals that contribute to taking control of the game. They also have fewer turnovers and mistakes, a sign of a team that plays a steady game and performs well.

Second, as for the difference between the groups depending on the sports class and game performance, the para-athlete's composition in each quarter depending on the sports class was as follows: the class of 13.92 points for 1 quarter, 13.91 points for 2 quarters, 13.88 points for 3 quarters, and 13.90 points for 4 quarters. As the medal group was compared with the other group, the average composition of the other group of para-athletes in quarters was higher than that of the medal group (1Q: 14 points, 2Q: 13.96 points, 3Q: 13.98 points, 4Q: 13.96 points) than the other group (1Q: 13.89 points, 2Q: 13.89 points, 3Q: 13.85 points, 4Q: 13.88 points), and the difference was significant. As to the difference between groups in sports performance, the difference in playing time between the medal group and the other group was in the order of 2.5 points (*p* = .001), 4.0 points (*p* = .001), 3.0 points (*p* = .002), and 1.5 points (*p* = .025). This shows that the difference in playing time was significant. In the medal group, the participation rate of players was even among different points. The class of 2.5 points participated in games about 4:30 min longer than the other group. The class of 2.5 points is considered significant since it can maintain the most stable posture among low classes of points and is highly capable of passing and shooting (29, 30).

**Figure 2:**
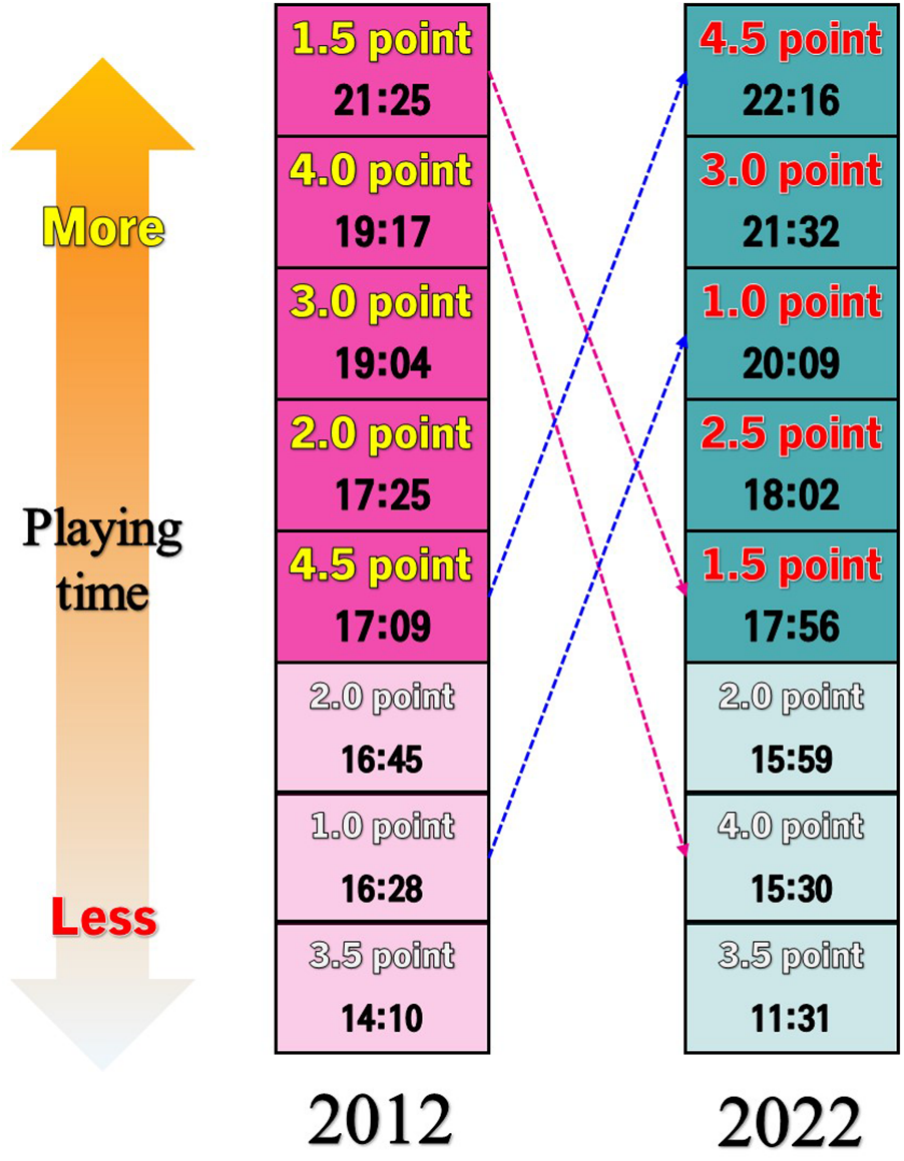
Comparison of playing time by sport class between 2012 and 2022.

Lastly, given the general trend in 2012 to 2022 international competitions, the sports class consideration in quarterly participation decreased. As to the playing time, depending on the range of points, that of 1.0 points, 3.0 points, and 4.5 points decreased while that of 1.5 points, 2.0 points, 3.5 points, and 4.0 points increased. Notably, the playing time of WB players of a mild case (4.5 points) decreased by about 4:21 min. In contrast, the playing time of players with 3.5 and 4.0 points increased as much as 2:39 min and 3:47 min, respectively. Notably, the playing time of the classes of 4.5 points and 1.0 points significantly decreased (see Figure 2).

**Figure 3:**
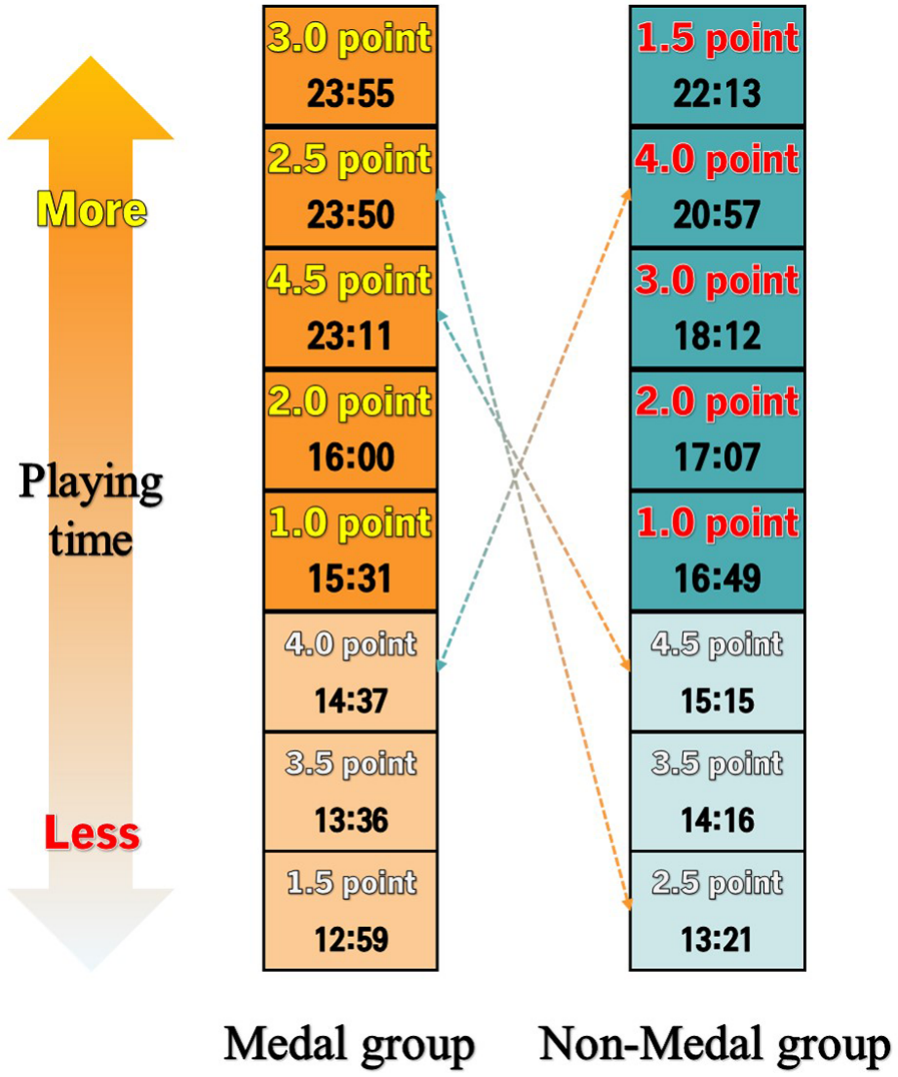
Comparison of playing time depending on the sport class in each group in 2022.

In WB, players of 4.5 points are of the mild case and play various roles in the team with relatively high motor abilities such as scoring and dribbling (29, 31). The playing time of players of 4.5 points decreased probably because the IWBF minimum disability criteria were revised in applying evidence-based sport classification as directed by the IPC after 2016 (31, 32). Among WB players attending the Tokyo PG held in 2021, sport classification was conducted again among players of 4.0 and 4.5 points. Except for 75% of the players who proved qualified among the 134, the rest had to undergo a reexamination. Some players with 4.5 points failed to meet the revised minimum disability criteria and thus could not attend the Tokyo PG (33). Further, players of 1.0 points have the most severe disability among WB players and, therefore, have limitations in wheelchair manipulation and moving speed. As WB advances, pursuing a fast pace has led to revisions. The roles of players of low points who had to play for less time were transferred to players of relatively high points, and the sports class of participant players was affected as a result (see Figure 3).

This study is significant because it examines the characteristics of WB games and analyzes trends in major game performance factors and sports class composition among major countries of excellent game performance, making visualized data available more efficiently and faster. It is expected that the findings of this study can be utilized effectively for game performance strategies that align with the most significant trend. In addition, this study will likely contribute to future studies on game performance in WB games. By analyzing the performance of WB and other para sports, this study aims to contribute to the growing field research supporting the development of these sports.

The limitations of this study are as follows. First, data were collected solely from official game records, and the analysis focused on objective factors centered around sport classification. Moreover, the scope of data collection was limited to the PG and WC, which restricted the range of available match data. Therefore, future studies should include position-specific analyses in addition to sport classifications, and explore key performance factors by incorporating data beyond official records—such as interviews with coaches involved in tactical decision-making. Furthermore, expanding the dataset to include recent tournaments and continental competitions would provide a more comprehensive understanding of performance in wheelchair basketball. In conclusion, we hope that future research will contribute to the development of effective strategies and efficient team compositions that reflect the unique characteristics of wheelchair basketball.

## 5 Conclusion

This study offers valuable insights for WB coaches to develop effective strategies and for players to enhance their performance by analyzing recent trends in sport classification and performance at the international level. The IPC continues to revise the classification system over specific periods to ensure fair competition and facilitate the participation of athletes with various types of impairments. As a result, countries are required to re-evaluate athlete classifications and select national representatives accordingly. This study confirmed that playing time varies across tournaments depending on the athletes' classifications, and this has a decisive impact on the selection of starting lineups. In high-performing countries, understanding the composition of classification points and their relation to playing time is a key factor in strategic planning.

## Data Availability

The original contributions presented in the study are included in the article/Supplementary Material, further inquiries can be directed to the corresponding author.
